# A Single V672F Substitution in the Spike Protein of Field-Isolated PEDV Promotes Cell–Cell Fusion and Replication in VeroE6 Cells

**DOI:** 10.3390/v11030282

**Published:** 2019-03-20

**Authors:** Asawin Wanitchang, Janya Saenboonrueng, Challika Kaewborisuth, Kanjana Srisutthisamphan, Anan Jongkaewwattana

**Affiliations:** Virology and Cell Technology Laboratory, National Center for Genetic Engineering and Biotechnology (BIOTEC), National Science and Technology Development Agency (NSTDA), Pathumthani 12120, Thailand; asawin.wan@biotec.or.th (A.W.); janya@biotec.or.th (J.S.); challika.kae@biotec.or.th (C.K.); kanjana.sri@ncr.nstda.or.th (K.S.)

**Keywords:** PEDV, spike, cell–cell fusion, syncytium, chimeric proteins, receptor preference

## Abstract

While porcine epidemic diarrhea virus (PEDV) infects and replicates in enterocytes lining villi of neonatal piglets with high efficiency, naturally isolated variants typically grow poorly in established cell lines, unless adapted by multiple passages. Cells infected with most cell-adapted PEDVs usually displayed large syncytia, a process triggered by the spike protein (S). To identify amino acids responsible for S-mediated syncytium formation, we constructed and characterized chimeric S proteins of the cell-adapted variant, YN144, in which the receptor binding domain (RBD) and S1/S2 cleavage site were replaced with those of a poorly culturable field isolate (G2). We demonstrated that the RBD, not the S1/S2 cleavage site, is critical for syncytium formation mediated by chimeric S proteins. Further mutational analyses revealed that a single mutation at the amino acid residue position 672 (V672F) could enable the chimeric S with the entire RBD derived from the G2 strain to trigger large syncytia. Moreover, recombinant PEDV viruses bearing S of the G2 strain with the single V672F substitution could induce extensive syncytium formation and replicate efficiently in VeroE6 cells stably expressing porcine aminopeptidase N (VeroE6-APN). Interestingly, we also demonstrated that while the V672F mutation is critical for the syncytium formation in VeroE6-APN cells, it exerts a minimal effect in Huh-7 cells, thereby suggesting the difference in receptor preference of PEDV among host cells.

## 1. Introduction

Porcine epidemic diarrhea virus (PEDV) causes severe enteric illness of swine (PED). The infection results in watery diarrhea, vomiting, and anorexia, which can lead to death in up to 100% of neonatal piglets [1,2]. Since its first appearance in the 1970s, PED outbreaks have caused enormous economic losses to the swine industry. Nowadays, outbreaks of PED are sporadically reported worldwide with emerging novel strains [3,4]. PEDV is an enveloped, non-segmented, positive-strand RNA virus, which constitutes the genus *Alphacoronavirus* of the family Coronaviridae. Its RNA genome encodes replicase proteins and structural proteins including spike (S), envelope (E), membrane (M), nucleocapsid (N), and an accessory protein (ORF3). The virus replicates efficiently in the enterocytes lining the villi of the small intestine, leading to cell death and severe villous atrophy [1].

While the replication of PEDV is thus far not completely understood, many assumptions have been made based on the data of well-characterized coronaviruses such as Severe Acute Respiratory Syndrome coronavirus (SARS-CoV), Middle East respiratory syndrome coronavirus (MERS-CoV), or transmissible gastroenteritis virus (TGEV). In particular, it has been shown that the structural proteins including S, M, and E proteins are gathered in the endoplasmic reticulum (ER) and transported to the endoplasmic reticulum–Golgi intermediate compartment (ERGIC), where they interact with the N protein-encapsidated viral genomes and assemble into viral particles followed by release via exocytosis of smooth-wall vesicles [5]. Coronavirus (CoV)-infected cells typically exhibit multinucleated giant syncytia triggered by the interaction of S at the cell surface and receptors of adjacent cells. S has been shown to be predominantly localized in the ERGIC or Golgi complex in cells transiently expressing S and M [6,7]. The interaction between S and M requires the ER retention signal (ERRS) comprising the tyrosine-dependent motif (Yxxϕ; ϕ is a hydrophobic residue) and the KxHxx motif at the C-terminus of S [8]. However, it remains largely unknown, in the context of infection, how S could escape the ER–Golgi retention and transit to the plasma membrane.

In general, cells infected with cell-adapted PEDV strains usually display large syncytia. However, those infected with early passaged PEDV strains or those freshly isolated from infected intestinal tissues rarely exhibited detectable syncytium formation [9,10]. In the current study, we investigate the ability to trigger cell–cell fusion by S derived from a poorly culturable isolate, G2, and that from a well-characterized cell-adapted strain, YN144, in the GII genogroup [11,12]. We then constructed various chimeric S constructs and evaluated cell–cell fusion in cells expressing each chimera. We could identify a key amino acid in the receptor binding domain (RBD) of S that plays a critical role in syncytium formation and growth in VeroE6-APN cells. Intriguingly, we also showed that S-mediated syncytium formation in Huh-7 cells was distinct from that in VeroE6 cells. The data presented here may provide more insights into the quest for PEDV receptors among various host cells.

## 2. Materials and Methods

### 2.1. Cells and Viruses

Human embryonic kidney cells (HEK293T, ATCC CRL-3216) and African green monkey kidney cells (VeroE6, ATCC CRL-1586) were maintained in Opti-MEM (ThermoScientific, Waltham, MA, USA), and human hepatocellular carcinoma cells (Huh-7, JCRB cell bank 0403) were cultured in Dulbecco’s Modified Eagle Medium (DMEM) low glucose (GE Healthcare Bio-Sciences, Pittsburg, PA, USA) at 37 °C with 5% CO_2_. All culture media were supplemented with 10% fetal bovine serum and an antibiotic/mycotic (ThermoScientific). Notably, VeroE6 cells stably expressing porcine aminopeptidase N (VeroE6-APN) were constructed by retroviral transduction as described previously [13]. Recombinant PEDVs used in this study were propagated in VeroE6-APN or Huh-7 cells, and virus titration was performed on VeroE6-APN or Huh-7 cell monolayers. Briefly, cells were grown to confluence in six-well plates, washed twice with 1 × Phosphate buffered saline (PBS), and inoculated with 10-fold serial dilutions of the recombinant PEDV. Infected cells were maintained in Opti-MEM containing recombinant trypsin (2 μg/mL) (ThermoScientific). At 24 h after infection, cells were fixed with 80% cold acetone for 10 min, washed twice with PBS, and blocked in PBS containing 10% fetal bovine serum(FBS) and 1% bovine serum albumin (BSA) for 1 h with gentle agitation. Subsequently, cells were incubated with mouse anti-PEDV N antibodies (Medgene, Brookings, SD, USA) and goat anti-mouse IgG alkaline phosphatase antibodies (Abcam, Cambridge, MA, USA). The plaque forming unit (PFU) was examined based on color formation after the addition of 1-Step™ NBT/BCIP Substrate Solution (ThermoScientific).

### 2.2. Plasmid Constructs

The full-length S of PEDV_YN144_ (S_YN144;_ GenBank KT021232.1) and PEDV_G2_ (S_G2_) were codon optimized for high expression in mammalian cells, synthesized, and cloned in the pUC57 cloning vector (Synbio Technologies, Monmouth Junction, NJ, USA). Of note, the amino acid sequence of S_G2_ is not yet available in the public database, but it is more than 99% identical to the strain 13JM-291 (GenBank ATJ03623). The synthetic gene was designed to be flanked by MluI and NotI restriction sites to facilitate the cloning into the modified pCAGGS expression plasmid. Removal of the C-terminal intracellular sorting motifs of the pCAGGS-S_YN144_ and pCAGGS-S_G2_ was carried out using site-directed mutagenesis by adding double stop codons before Yxxϕ and KxHxx motifs at the cytoplasmic tail. Notably, to facilitate the construction of chimeric S, unique restriction enzyme cutting sites (NheI and SmaI) were designed to swap the fragment spanning residues 440–940 between S_YN144_ and S_G2_.

The full-length infectious clones of PEDVmCherry bearing designated S constructs were generated using a strategy described previously [13]. Briefly, a cloning vector (pTZ-GH) expressing S–E–M of the prototype PEDV_AVCT12_ were constructed and used as an intermediate plasmid. Each S construct was introduced into the pTZ-GH by replacing the original S using In-Fusion HD Cloning (Clontech, Mountain View, CA, USA) following the manufacturer’s instruction. The full-length pSMART-BAC-PEDVmCherry bearing each S was then constructed by replacing the PacI–MluI fragment of the pTZ-GH with that of the infectious cDNA clone.

### 2.3. Syncytium Formation Assay

VeroE6-APN or Huh-7 cells (5 × 10^5^ cells/mL) were seeded in a six-well plate in Opti-MEM or DMEM low glucose supplemented with 10% FBS. At 24 h, cells were washed with 1 × PBS and maintained in media without FBS. Cells were transfected with pCAGGS expressing S (1.5 µg) using FuGENE HD (Promega, Madison, WI, USA) according to the manufacturer’s instructions. Media containing transfection mixture were removed 8 h post transfection (hpt). Cells were subsequently maintained in media containing trypsin (2 μg/mL). At 24 hpt, cells were examined under an inverted light microscope for syncytium formation.

### 2.4. Recovery of Recombinant PEDV from Infectious cDNA Clones

HEK293T cells were grown to 90–95% confluence and transfected with 2 µg of each infectious cDNA clone construct using FuGENE HD. No trypsin was added at this step to avoid cell toxicity. At 72 hpt, supernatants were adsorbed onto the monolayer of VeroE6-APN or Huh-7 cells and cultured in Opti-MEM supplemented with trypsin (2 μg/mL). Cells were monitored daily for syncytium formation and mCherry expression under a fluorescence microscope. Supernatants were harvested when cytopathic effects were apparent. Virus supernatant suspensions were stored at −80 °C.

### 2.5. Immunofluorescence Assay

VeroE6-APN cells were grown on coverslips in six-well plates and transfected with pCAGGS expressing S. At 24 hpt, cells were washed with PBS and fixed with 4% paraformaldehyde (PFA) for 20 min at 4 °C. Cells were then washed and blocked with PBS containing 10% FBS, 1% BSA for 1 h for surface staining. For intracellular staining, 0.2% TritonX-100 was also included in the blocking solution. Cells were subsequently incubated for 1 h with mouse anti-PEDV S1 antibodies (a kind gift from Dr. Qigai He) in 10% FBS at a dilution of 1:500. After washing, goat anti-mouse IgG Alexa Fluor 647 (Abcam) in 10% FBS at a dilution of 1:1000 was added and further incubated for 1 h. The glass slips were mounted on slides with ProLong Gold Antifade Mountant with DAPI (ThermoScientific). The samples were analyzed by Fluoview^TM^ FV1000 confocal microscopy (Olympus, Tokyo, Japan).

### 2.6. Flow Cytometry

HEK293T cells were seeded in six-well plates and transfected with pCAGGS expressing PEDV S (1.5 µg). At 24 hpt, cells were carefully detached, fixed with 4% PFA, and blocked in blocking buffer (2% FBS and 0.5% BSA in PBS) for 1 h. Cells were then incubated with mouse anti-PEDV S1 antibodies for 1 h. An anti-calreticulin ER marker (Abcam) was used as a non-permeabilized condition control. Cells were washed with PBS and incubated with anti-mouse Alexa Fluor 647 (IgG H + L) antibodies for 1 h. Cells were washed and analyzed on a FlowSight^®^ Imaging Flow Cytometer (Merck, Kenilworth, NJ, USA).

### 2.7. Western Blot Analysis

Cells were lysed with mammalian cell lysis buffer (50 mM Tris HCl pH 8.0, 100 mM NaCl, 2 mM DTT, 5 mM EDTA, 0.5% NP-40, and protease inhibitors). Aliquots of whole cell lysates were separated by sodium dodecyl sulfate polyacrylamide gel electrophoresis (SDS-PAGE), and the proteins were then transferred onto nitrocellulose membranes (Bio-Rad Laboratories, Hercules, CA, USA). The membrane was blocked in 5% dry powdered milk and incubated with mouse anti-PEDV S1 or −β actin antibodies. The membranes were then incubated with horseradish peroxidase (HRP)-conjugated goat anti-mouse IgG (1:5000; Biolegend, San Diego, CA, USA). Target proteins were visualized using Clarity Western ECL Substrate (Bio-Rad Laboratories).

### 2.8. Statistical Analysis

GraphPad Prism 7.0 (GraphPad Software Inc., La Jolla, CA, USA) was used for statistical analyses.

## 3. Results

### 3.1. Distinct Cell–Cell Fusion Induced by S_YN144_ and S_G2_ in VeroE6-APN Cells

**Figure 1:**
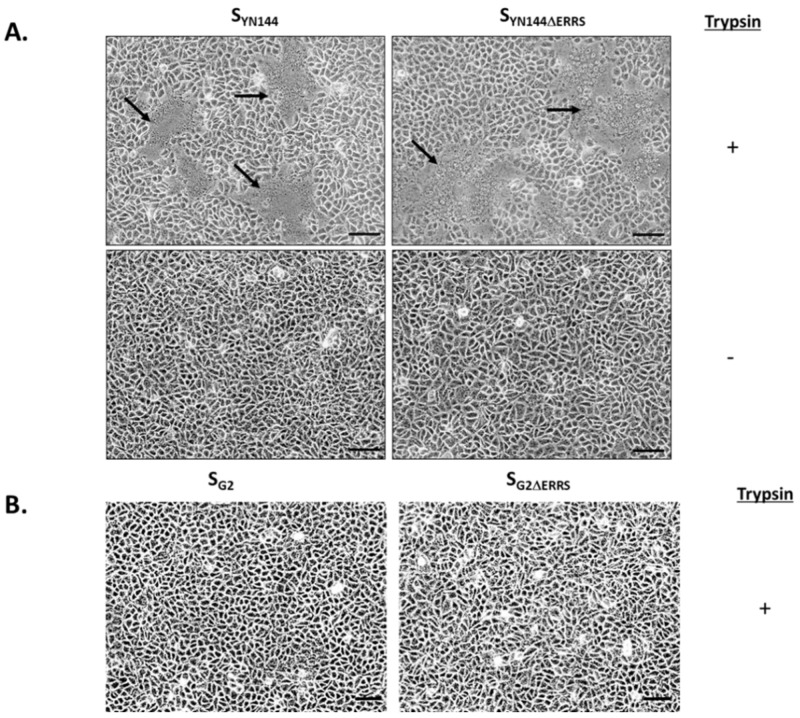
Formation of syncytia in VeroE6 cells stably expressing porcine aminopeptidase N (VeroE6-APN) expressing porcine epidemic diarrhea virus (PEDV) S derived from YN144 and G2. (**A**) VeroE6-APN cells were transfected with pCAGGS expressing S_YN144_ with or without the C-terminal motifs (S_YN144ΔERRS_). Cells were cultured in the presence or absence of trypsin (2 µg/mL). Syncytium formation was evaluated under a light microscope at 24 h post transfection (hpt). Arrows denote syncytium formation. Scale 100 µm. (**B**) VeroE6-APN cells were transfected with pCAGGS expressing S_G2_ with or without the C-terminal motifs (S_G2ΔERRS_). Cells were cultured in the presence of trypsin (2 µg/mL), and the syncytium formation was assessed at 24 hpt. Scale 100 µm.

PEDV strain YN144 is a well-characterized cell-adapted PEDV in the GII genogroup. Not only does it replicate efficiently in mammalian cells, but it also exhibits a highly syncytial phenotype in cell culture [11,12]. While the C-terminal motifs are believed to play a key role in its ability to trigger cell–cell fusion [14,15,16,17], it is important to note that S_YN144_ harbored no change of amino acids at the C-terminal region upon multiple passages [12]. To confirm the ability of S_YN144_ in syncytium induction, we synthesized codon-optimized S_YN144_, cloned into the pCAGGS expression vector and evaluated the cell–cell fusion activity in transfected VeroE6-APN cells. Notably, codon-optimization is required to obtain a detectable expression of CoV S in transfected cells [18]. As depicted in Figure 1A, cells transfected with pCAGGS expressing S_YN144_ exhibited clear syncytium formation in the presence of trypsin. Without trypsin treatment, no syncytia were observed (Figure 1A). We subsequently removed the C-terminal motifs from the S_YN144_ (S_YN144 ΔERRS_) and evaluated the syncytium formation in transfected VeroE6-APN cells in the presence and absence of trypsin. The absence of the C-terminal motifs resulted in the induction of notably larger syncytium formation in transfected cells (Figure 1A). In contrast to S_YN144_, when we transfected VeroE6-APN cells with pCAGGS expressing S_G2_, we could not detect any syncytia even in the presence of trypsin or absence of the C-terminal motifs (Figure 1B). Of note, the expression level of S_G2_ and S_YN144_ were shown previously to be comparable [9], thereby suggesting that the loss of cell–cell fusion activity was not due to the difference in protein expression. These results suggest that S derived from the cell-adapted variant can trigger syncytium formation regardless of the C-terminal motifs, corroborating the notion that changes during serial passages may enable the S protein to trigger cell–cell fusion in cultured cells.

### 3.2. S_G2_ Lacking the C-Terminal Motifs Showed Strong Cell Surface Expression

**Figure 2:**
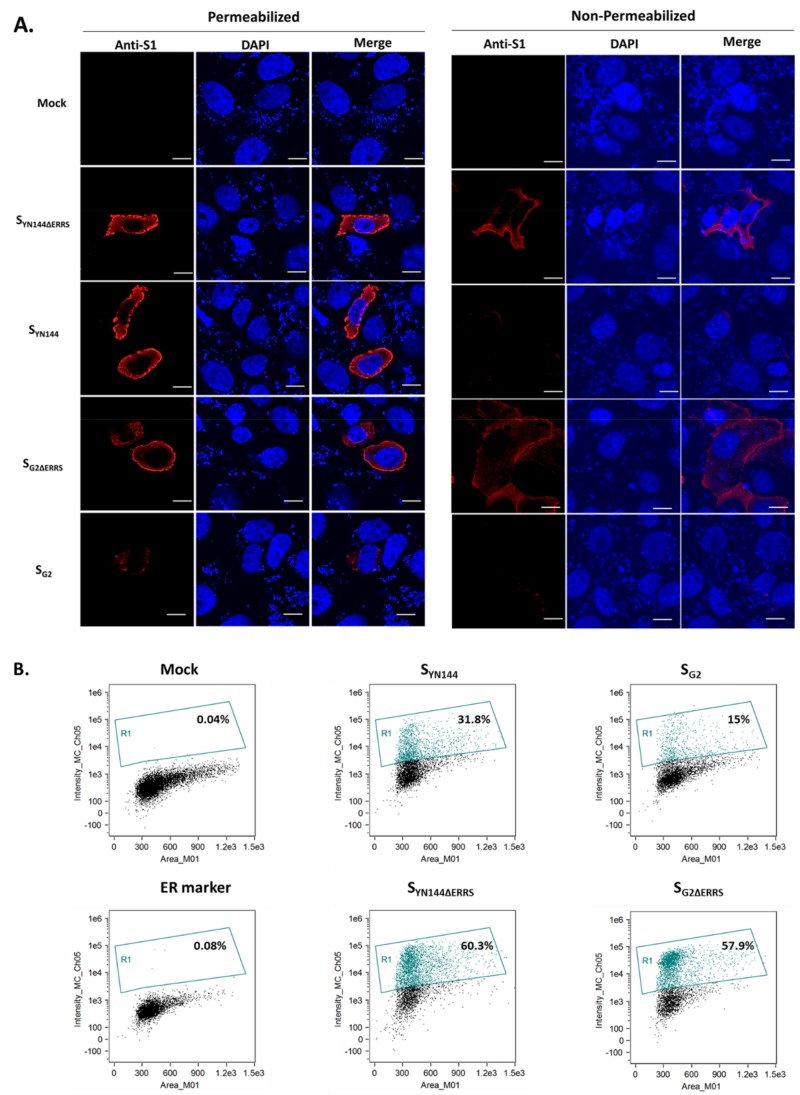
Surface expression of PEDV S lacking the C-terminal motifs. (**A**) VeroE6-APN cells were transfected with pCAGGS expressing indicated PEDV S. At 24 hpt, cells were subjected to immunofluorescence analysis under permeabilized and non-permeabilized conditions using mouse anti-PEDV S1 and goat anti-mouse IgG Alexa Fluor 647 (IgG H + L) antibodies as primary and secondary antibodies. The glass slips were mounted on slides with DAPI. The samples were analyzed by confocal microscopy. Scale 5 µm. (**B**) HEK293T cells were transfected with pCAGGS expressing indicated PEDV S. At 24 hpt, cells were detached, fixed, and incubated with mouse anti-PEDV S1 monoclonal antibodies. Cells were subsequently incubated with goat anti-mouse Alexa Fluor 647 (IgG H+L) antibodies and analyzed on a flow cytometer. An anti-calreticulin endoplasmic reticulum (ER) marker was used as a non-permeabilized condition control.

The finding that S_G2ΔERRS_ failed to induce syncytium formation in transfected cells prompted us to speculate whether the protein expression at the cell surface, which is required for cell–cell fusion, was disturbed by this modification. To this end, we transfected VeroE6-APN cells and assessed the surface expression of S_G2ΔERRS_ by confocal microscopy. As a comparison, S_YN144ΔERRS_ was also investigated. We found that both S_G2ΔERRS_ and S_YN144ΔERRS_ displayed comparably strong surface expression (Figure 2A). Surprisingly, despite the apparent syncytium formation, the inclusion of the C-terminal motifs to S_YN144_ resulted in a substantial decrease in surface expression (Figure 2A). Moreover, S_G2_ containing the intact C-terminal motifs (S_G2_) also showed hardly detectable surface expression (Figure 2A), confirming the role of ERRS in S cellular trafficking. To further confirm the results obtained from the immunofluorescence assay (IFA), we employed flow cytometry to assess the surface expression of S_G2ΔERRS_. As expected, the absence of the C-terminal motifs could significantly augment the surface expression of both S_G2_ and S_YN144_ (Figure 2B). The fact that S_G2ΔERRS_ showed strong surface expression but could not trigger detectable syncytium indicates that surface expression alone may not be sufficient to trigger cell–cell fusion.

### 3.3. Chimeric S_YN144_ Bearing RBD and S1/S2 Cleavage of S_G2_ Failed to Induce Syncytium Formation

**Figure 3:**
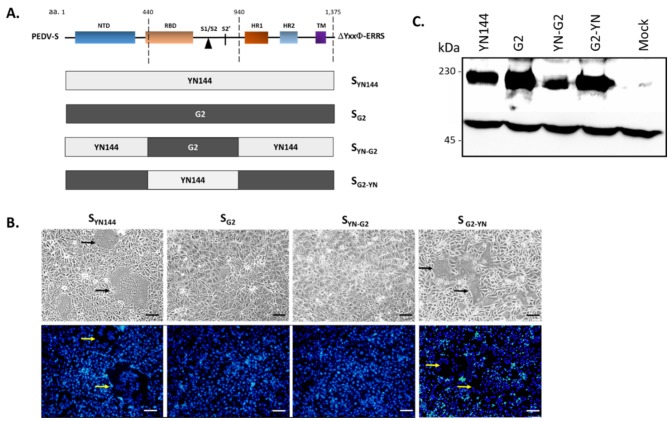
Syncytium formation in VeroE6-APN cells expressing chimeric S_YN144_ bearing receptor binding domain (RBD) and S1/S2 cleavage of S_G2_. (**A**) Schematic representation of chimeric PEDV S in which the RBD and the S1/S2 cleavage site of S_YN144_ were replaced with that of S_G2_ or vice versa. (**B**) VeroE6-APN cells were transfected with pCAGGS expressing S_YN144_, S_G2_, or the chimeric S_YN-G2_ and S_G2-YN_ and cultured in the presence of trypsin. At 24 hpt, cells were evaluated for syncytium formation. Hoechst was used to stain nuclei. Arrows denote the formation of the syncytium. Scale 100 µm. (**C**) Western blot analysis of HEK293T cells transfected with pCAGGS expressing S_YN144_, S_G2_, S_YN-G2_, or S_G2-YN_. Mouse anti-PEDV S1 and –*β* actin antibodies were used to detect protein expression in cell lysates harvested 48 hpt.

Successful syncytium formation requires not only interaction of S with the receptor, but also proteolytic cleavage of S to facilitate cell–cell fusion [19]. We thus sought to determine whether the RBD and the S1/S2 cleavage site (S1/S2) contribute to the syncytium induction activity of S_G2_ and S_YN144_. To this end, we constructed the chimeric S_YN144_ harboring the RBD and S1/S2 derived from S_G2_ (S_YN-G2_) as well as the S_G2_ carrying the RBD and S1/S2 of S_YN144_ (S_G2-YN_) (Figure 3A). Of note, to rule out the effect of surface localization, all constructs were devoid of the C-terminal motifs. When each construct was evaluated for its ability to trigger syncytia in transfected cells, we found that while S_YN144_ triggered large syncytia, S_YN-G2_ failed to do likewise, similar to that in cells transfected with S_G2_ (Figure 3B). In contrast, we detected clear syncytium formation, albeit relatively smaller than those of S_YN144_, in cells transfected with S_G2-YN_ (Figure 3B). Notably, the absence of syncytia in cells expressing S_YN-G2_ and S_G2_ was not due to a defect in protein expression, as the western blot analysis showed relatively high protein expression of all constructs (Figure 3C). Taken together, these results suggest that amino acids in the RBD and S1/S2 of PEDV S could influence S-mediated induction of cell–cell fusion in VeroE6-APN cells.

### 3.4. Amino Acids in the RBD Are Critical for Cell–Cell Fusion Induced by S_YN144_

**Figure 4:**
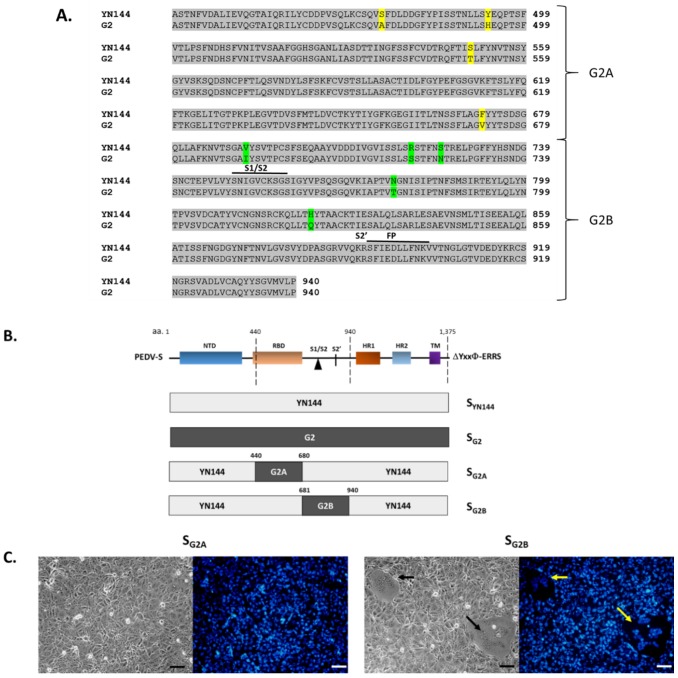
RBD plays a key role in the syncytium formation in VeroE6-APN cells. (**A**) Amino acid alignment between S_YN144_ and S_G2_ in the region covering the RBD and S1/S2 cleavage site. Colored residues are those that are different between the two strains. (**B**) Schematic representation of chimeric PEDV S in which the RBD or the S1/S2 cleavage site of S_YN144_ was replaced with that of S_G2_. (**C**) VeroE6-APN cells were transfected with pCAGGS expressing the chimeric S_G2A_ and S_G2B_ and cultured in the presence of trypsin. At 24 hpt, cells were evaluated for syncytium formation. Hoechst was used to stain nuclei. Arrows denote the formation of the syncytium. Scale 50 µm.

Both S_YN144_ and S_G2_ are derived from the GIIb genogroup, and the RBD and S1/S2 between the two proteins differ by nine amino acids with four residues in the RBD and five residues in the S1/S2 (Figure 4A). We further constructed and examined the syncytium-inducing property of chimeric S variants, in which RBD and S1/S2 of S_YN144_ were replaced with those of S_G2_, designated S_G2A_ and S_G2B_ (Figure 4B). As depicted in Figure 4C, we found that VeroE6-APN cells transfected with S_G2B_ but not S_G2A_ exhibited extensive syncytium formation, thereby suggesting that the RBD of S_G2_ could render S_YN144_ unable to induce cell–cell fusion.

**Figure 5:**
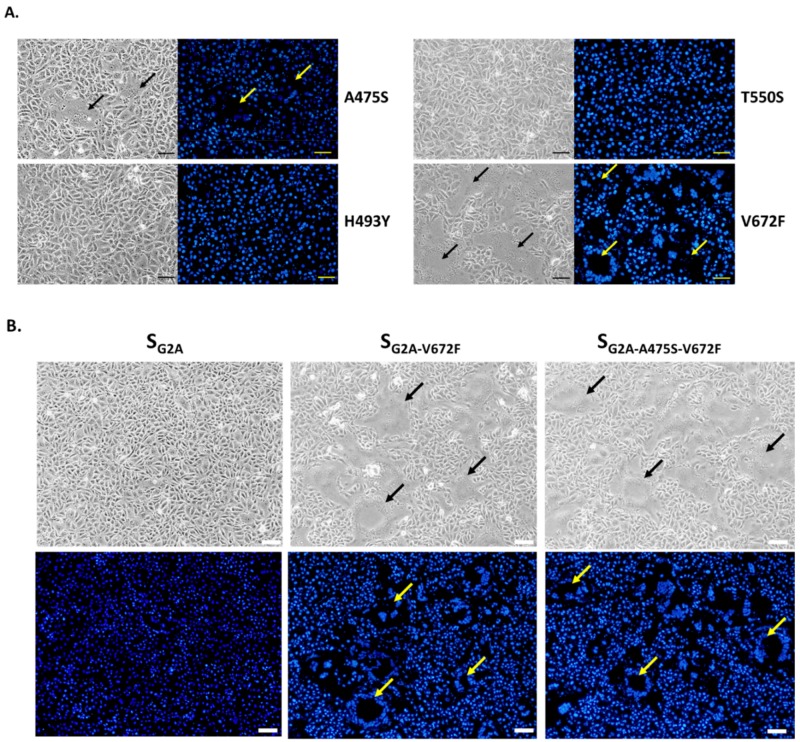

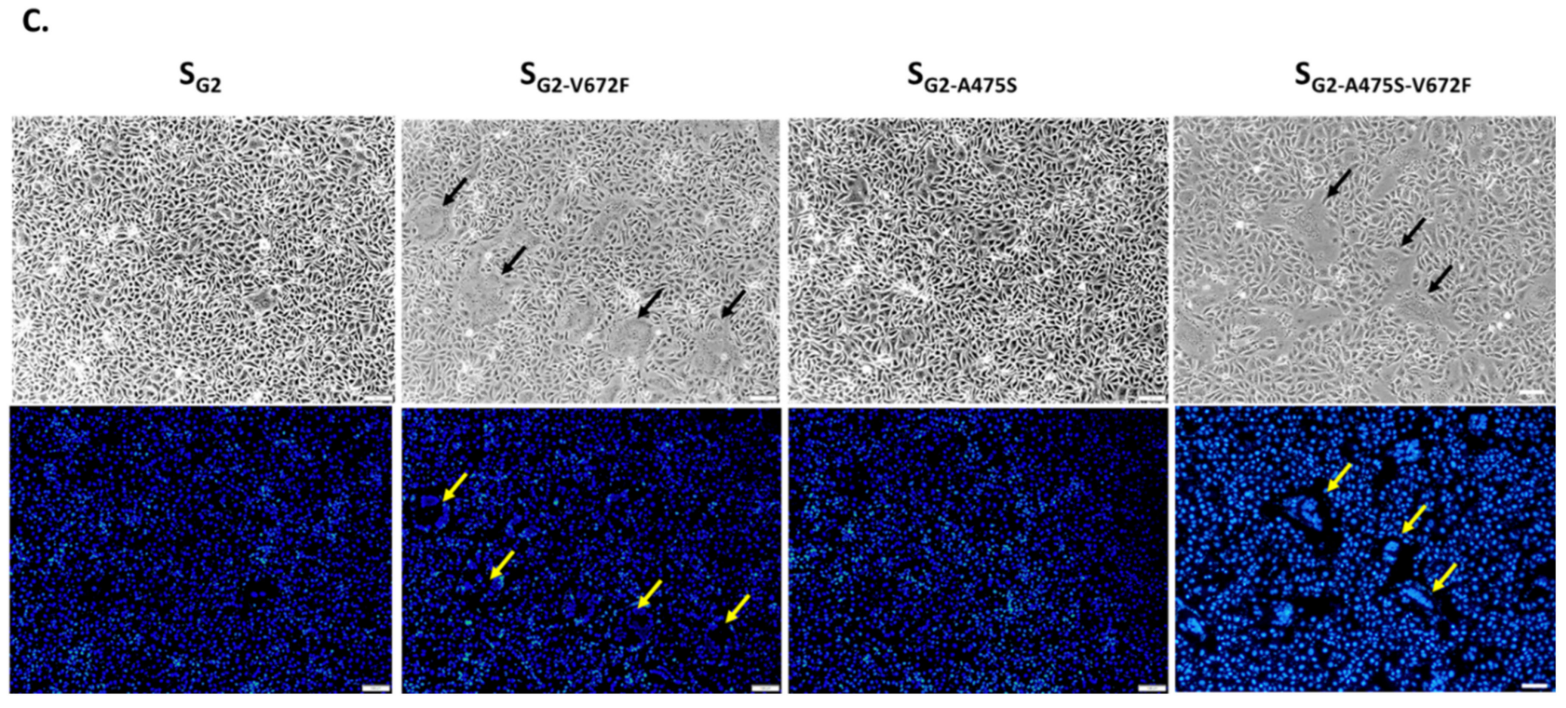
Effect of amino acid substitution in the RBD of S_G2_ on syncytium formation in VeroE6-APN cells. (**A**) VeroE6-APN cells were transfected with pCAGGS expressing S_G2A_ with single amino acid substitution at positions 475, 493, 550, and 672 and treated with trypsin. At 24 hpt, cells were assessed for syncytium formation. Scale 50 µm. (**B**) VeroE6-APN cells were transfected with pCAGGS expressing S_G2A_, S_G2A-V672F_, and S_G2A-A475S-V672F_ and cultured in the presence of trypsin. At 24 hpt, cells were evaluated for syncytium formation. Hoechst was used to stain nuclei. Arrows denote the formation of the syncytium. Scale 100 µm. (**C**) VeroE6-APN cells were transfected with pCAGGS expressing wild-type S_G2_, S_G2-V672F_, S_G2-A475S_, and S_G2-A475S-V672F_ and cultured in the presence of trypsin. At 24 hpt, cells were evaluated for syncytium formation. Hoechst was used to stain nuclei. Arrows denote the formation of the syncytium. Scale 100 µm.

We next sought to determine the effect of each amino acid in the RBD of S_G2_ by introducing a mutation of each amino acid in the RBD of S_G2B_ (A475S, H493Y, T550S, and V672F) and assessed the presence of syncytia in cells transfected with each construct. As shown in Figure 5A, cells expressing S_G2A_ carrying H493Y and T550S did not display detectable syncytium formation. In contrast, those expressing S_G2A_ with A475S and V672F exhibited detectable syncytia. Among all mutations, cells expressing S_G2A_ with V672F mutation showed remarkably large syncytia, suggesting that F at the position 672 is critical for the syncytium formation in VeroE6-APN cells. Furthermore, when cells expressing S_G2A_ carrying double mutations (A475S, V672F) were assessed for syncytium formation, we observed syncytia slightly larger than those with a single V672F substitution (Figure 5B). These data altogether point to the possibility that A475S might synergize with V672F in promoting cell–cell fusion. To investigate the importance of the F672 residue, we introduced a single V672F substitution to S_G2_ (S_G2-V672F_) and evaluated its ability to induce cell–cell fusion. While the wild-type S_G2_ could not trigger detectable syncytium in transfected cells, we could detect large syncytia in VeroE6-APN cells expressing S_G2-V672F_ (Figure 5B). Notably, cells expressing S_G2_ with a single A475S mutation (S_G2-A475S_) did not show detectable syncytium formation. However, when double mutations (A475S, V672F) were introduced, large syncytia were observed (Figure 5C). It is also important to note that syncytia in S_G2-V672F_-transfected cells were smaller than those transfected with S_G2A-V672F_ (Figure 5B), thereby further supporting the notion that other amino acids in the S_YN144_ might work in concert with V672F to drive the optimal cell–cell fusion.

### 3.5. The Effect of the V672F Substitution Is Specific for VeroE6 Cells

**Figure 6:**
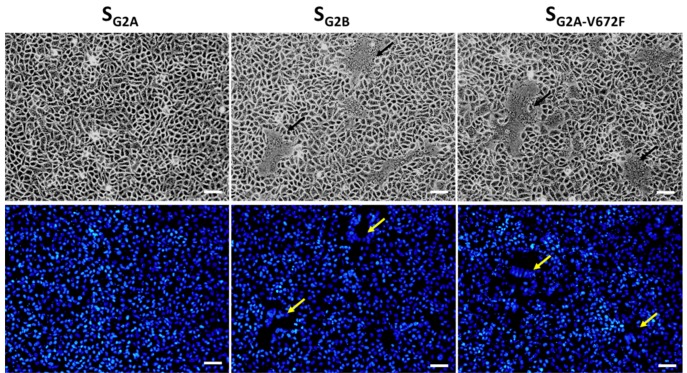
Syncytium formation in VeroE6 cells expressing PEDV S. VeroE6 cells were transfected with pCAGGS expressing S_G2A_, S_G2B_, and S_G2A-V672F_ and treated with trypsin. At 24 hpt, cells were assessed for syncytium formation. Hoechst was used to stain nuclei. Arrows denote the formation of the syncytium. Scale 50 µm.

The fact that residues in the RBD are critical for cell–cell fusion strongly suggests that the interaction of S and the receptor is critical for the process. While porcine aminopeptidase N (pAPN) was initially identified as a natural receptor of PEDV [20,21,22], several recent studies demonstrated that neither the overexpression, nor the complete knockout of APN affects PEDV infectivity in cultured cells [23,24,25]. Since our results were based on the transfection of plasmids in VeroE6-APN cells, we asked whether the expression of chimeric S in the parental VeroE6 cells would result in syncytium formation in a similar manner. To this end, we transfected pCAGGS expressing S of various constructs into parental VeroE6 cells and examined the syncytium formation. As shown in Figure 6, similar to what was observed in VeroE6-APN cells (Figure 4 and Figure 5), VeroE6 cells displayed large syncytium formation when transfected with the plasmid expressing S_G2B_ and S_G2A-V672F_. In contrast, those transfected with the plasmid expressing S_G2A_ showed undetectable syncytium formation. These data suggest that PEDV S likely binds to the receptor present in both VeroE6 and VeroE6-APN cells.

**Figure 7:**
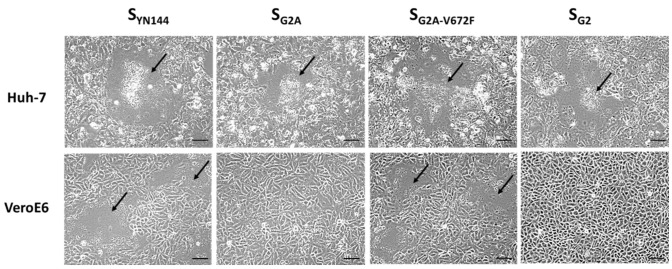
Syncytium formation in Huh-7 and VeroE6 cells expressing PEDV S. Huh-7 and VeroE6 cells were transfected with pCAGGS expressing S_YN144_, S_G2A_, S_G2A-V672F_, and S_G2._ Transfected cells were cultured in the presence of trypsin. At 24 hpt, cells were examined for syncytium formation. Arrows denote the formation of the syncytium. Scale 50 µm.

It is currently unknown how PEDV initiates its entry into Vero or VeroE6 cells. The receptor residing on the VeroE6 cell surface also remains to be identified. Indeed, the fact that the V672F substitution could enhance S-mediated cell–cell fusion in VeroE6 cells points to the possibility that this residue might be important for the interaction of S and its receptor. To test whether V672F is specific for the receptor of VeroE6 cells, we transfected VeroE6 and Huh-7 cells with plasmids expressing various constructs of S and compared syncytium formation in both cell lines. To our surprise, large syncytia could be detected in Huh-7 cells transfected with all chimeric S constructs including the S_G2A_ that could not trigger cell–cell fusion in VeroE6 cells (Figure 7). Interestingly, we also found syncytium formation in Huh-7 cells transfected with pCAGGS expressing S_G2_. These results suggest that the mode of binding between S and receptors on VeroE6 cells may be different from that of Huh-7 cells.

### 3.6. Effect of the V672F Substitution on the Recombinant PEDV Growth

**Figure 8:**
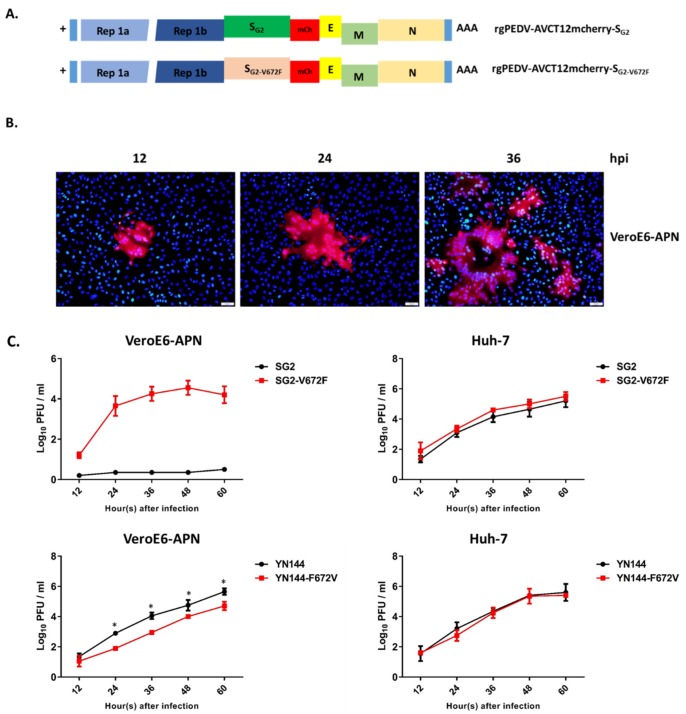
Effect of the V672F substitution on the growth of PEDV in VeroE6-APN cells. (**A**) Schematic representation of infectious clones used for generation of recombinant PEDV bearing S_G2_ or S_G2-V672F_. (**B**) VeroE6-APN cells were adsorbed with supernatants harvested from HEK293T cells transfected with the infectious clone of rgPEDV-AVCT12mCherry-S_G2-V672F_. The expression of mCherry was assessed by fluorescence microscopy at indicated times after adsorption. Scale 50 µm. (**C**) Growth kinetics of recombinant PEDV bearing S_G2_, S_G2-V672F_, S_YN144_, and S_YN144_ with F672V substitution in VeroE6-APN and Huh-7 cells. Error bars represent mean ± SD. * *p* < 0.05.

To assess the effect of the V672F substitution in the virus infection context, we modified the infectious clone of PEDV_AVCT12_ carrying the mCherry gene (rgPEDV-AVCT12mCherry) [13] by swapping the original S with S_G2_ and with that harboring the V672F mutation (Figure 8A). We subsequently rescued the viruses and assessed their growth in VeroE6-APN and Huh-7 cells. As expected, while PEDV with the wild-type S_G2_ grew poorly with undetectable syncytia (data not shown), the virus carrying the V672F mutation showed clear syncytium formation during the first passage in VeroE6-APN cells (Figure 8B). The replication kinetics of the recombinant virus also correlated with the ability to form syncytia in infected cells (Figure 8C). Notably, both recombinant viruses could grow in Huh-7 cells with comparable growth kinetics (Figure 8C). Interestingly, the recombinant virus bearing S_YN144_ with F672V exhibited significantly slower growth kinetics when compared to those with the wild-type S_YN144_ in VeroE6-APN but not in Huh-7 cells (Figure 8C). Taken together, these data suggest that V672F could enable S_G2_ to possibly bind to its receptor, undergo cell–cell fusion, and initiate replication in VeroE6-APN cells.

## 4. Discussion

In vitro propagation of the field-isolated PEDV has been a technical challenge since the virus was first isolated decades ago [26]. Through multiple passaging in cultured cells, cell-adapted PEDV strains could occasionally be obtained. Although cells infected by cell-adapted PEDV usually display extensive syncytia, those infected by early passaged PEDV strains or in vivo exhibit minimal, if not undetectable, syncytium formation. While it is clear that changes in the S protein accumulated during consecutive passages in Vero cells are associated with the ability of S to induce large syncytium formation, the mechanism by which these mutations in the S protein affect the cell–cell fusion and virus growth in the host cells is still not well understood. In this study, we investigated the ability of PEDV S to induce syncytium formation in VeroE6 cells using those that derived from a field isolate (G2) and a cell-adapted variant (YN144).

The syncytium formation is usually triggered as a result of the binding between S on the infected cells’ surface and its receptor on adjacent cells. However, it is not clear how S, especially of cell-adapted strains, could transit to the plasma membrane as its cellular localization is known to be tightly regulated by the C-terminal motifs [5]. A few cell-adapted strains in the GI genogroup such as SM98, AVCT12, or CHM2013 or GII genogroup such as FL2013 could form large syncytia in infected cells mainly due to the natural C-terminal deletion of the S gene [13,27,28]. Interestingly, Vero cells infected with some cell-adapted strains, such as YN144 [12], PT-P96 [29], or KNU-141112 [30], could exhibit large syncytia even though the viruses still possess the intact C-terminal motifs. We showed here that S_YN144_ displayed hardly detectable cell surface expression, while S_YN144ΔERRS_ showed strong surface expression in transfected cells (Figure 2A). These data thus suggest that a minute amount of the S protein on the cell surface might be sufficient for the induction of syncytium formation. It is also important to note that S_G2ΔERRS_, though it displayed strong surface expression, could not form detectable syncytium in VeroE6-APN cells (Figure 2A), which further supports the notion that the surface expression is not sufficient for promoting syncytium formation in cells expressing PEDV S.

In addition, results from this study lead us to speculate that regions outside the C-terminal motifs might play a role in the S-mediated formation of syncytium in VeroE6 cells. Syncytium formation in cells expressing various chimeric S constructs indeed revealed that the RBD of S_YN144_, but not the S1/S2 junction or fusion peptide cleavage site, might be critical for the cell–cell fusion in VeroE6-APN cells. Specifically, a single amino acid substitution at position 672 in the RBD from valine to phenylalanine (V672F) could render PEDV S of a typical field isolate to mediate cell–cell fusion in VeroE6-APN cells. Moreover, the recombinant PEDV bearing S_G2_ with V672F mutation also replicated efficiently in VeroE6-APN cells, while that harboring the wild-type S_G2_ grew poorly, thereby supporting the notion that the syncytium formation is likely associated with the high-growth characteristic of PEDV. It should be emphasized, however, that V672F was specific for syncytium induction in VeroE6 or VeroE6-APN cells. We showed that SY_N144_ carrying the RBD derived from S_G2_ could effectively induce syncytium in Huh-7, but not in VeroE6-APN cells. This finding thus points to the possibility that S might interact with different receptors on Huh-7 and VeroE6-APN cells. Given that pAPN has been recently shown to be unnecessary for PEDV entry [22,23,25], the functional PEDV receptor on Huh-7 cells is unlikely to be the human APN but may instead be some other surface protein(s) that might be absent in Vero or VeroE6 cells. Another important point worth mentioning is that all recombinant viruses that grew poorly in VeroE6-APN cells could replicate efficiently in Huh-7 cells. These results suggest that Huh-7 cells might be a more suitable host for isolating and propagating field-isolated PEDV than Vero or VeroE6 cells.

Sequence analyses of PEDV S in the GenBank database revealed that the majority amino acid at the position 672 of GII PEDV S is valine (V672) with less than 5% of available data showing phenylalanine (F672) and isoleucine (I672). Interestingly, F672 is also found in an early passage of YN144, YN15 (GenBank KT021228.1), which indicates that this specific residue is unlikely to be the result of adaptive mutations that occurred during serial passages. To the best of our knowledge, V672F has never been reported specifically as a mutation in cell-adapted strains. One cell-adapted strain, so-called LZW, was reported to carry F672 at passage 24 (GenBank AIJ01341), but it is not known whether this residue was also present in the original isolate. It is notable that while several cell-adapted PEDVs have been recently characterized and reported, common mutations in S that confer cell adaptation are not yet identified. For example, Zhou et al. have recently reported that A389S, P803H, Q825H, I1,010T, and C1,362G mutations in S of PEDV strain ZJ15XS0101 at passage 120 could enable the virus to replicate up to 10.125 log10 TCID50/mL in VeroE6 cells [31]. However, the same mutations were never reported in other known cell-adapted strains. We also demonstrated in this study that the recombinant virus bearing V672 showed slower growth kinetics than that with F672 in VeroE6-APN cells. It is thus likely that PEDV with F672 could be found in some natural isolates and these strains might be more efficiently propagated in Vero or VeroE6 cells when compared to those carrying S with V672.

In summary, using the in vitro assay to assess S-mediated cell–cell fusion and the reverse genetics of PEDV, our results altogether suggest that syncytium formation is associated with the high growth characteristic of cell-adapted PEDV. We demonstrate in this study that a single V672F substitution could render PEDV carrying S derived from a poorly culturable isolate to trigger large syncytia and replicate efficiently in VeroE6 cells. Exactly how V672F substitution causes S-mediated cell–cell fusion is still not known. However, our data point to the possibility that this single mutation in the RBD might lead to a more favorable interaction between S and its host cell-specific receptor. Further functional analyses are needed to elucidate the detailed mechanism of how V672F contributes to PEDV S-mediated cell–cell fusion. More importantly, the identification of the PEDV receptor on VeroE6 cells will provide further insights.
